# Supplementary material for: Heterologous expression and characterization of synthetic polyester-degrading cutinases from Fusarium spp. in Aspergillus niger
Source: Microbiol Spectr. 2025 Oct 24;13(12):e02177-25. doi: 10.1128/spectrum.02177-25 (PMC12671159; doi:10.1128/spectrum.02177-25)
Supplement: Supplemental material — Data S1 to S4; Tables S1 to S5; Fig. S1. [file spectrum.02177-25-s0001.pdf]

Supplemental data 1

Alignment FoCut 5 with Cut 5 and the reference Cutinases (derived via ITS sequencing and NCBI)

```
FFUJ_10428_cut3     MKFSIISTLFAATASALPAGQDAAALEARQLGGSITRNDLANGNSGSCPGVIFIYARGST      60
Cut5_#62            MKFSIISTLLAATASALPAGQDAAALEARQLGGSITRNDLANGNSGSCPGVIFIYARGST      60
FOZG_16529_cut3     MKFSIISTLLAATASALPAGQDAAALEARQLGGSITRNDLANGNSGSCPGVIFIYARGST      60
Cut5_#38            MKFSIISTLLAATASALPAGQDAAALEARQLGGSITRNDLANGNSGSCPGVIFIYARGST      60
FoCut5              ----------------LPAGQDAAALEARQLGGSITRNDLANGNSGSCPGVIFIYARGST      44
                                    ********************************************

FFUJ_10428_cut3     EAGNLGTLGPRVASKLEAKYGKNGVWIQGVGGAYRATLGDNALPRGTSSAAIREMLGHFN      120
Cut5_#62            EAGNLGTLGPRVASQLEAKYGRNGVWIQGVGGAYRATLGDNALPRGTSSAAIREMLGHFN      120
FOZG_16529_cut3     ESGNLGTLGPRVASKLEAKYGKNGVWIQGVGGAYRATLGDNALPRGTSSAAIREMLGHFN      120
Cut5_#38            ESGNLGTLGPRVASKLEAKYGKNGVWIQGVGGAYRATLGDNALPRGTSSAAIREMLGHFN      120
FoCut5              ESGNLGTLGPRVASKLEAKYGKNGVWIQGVGGAYRATLGDNALPRGTSSAAIREMLGHFS      104
                    *:************:******:*************************************.

FFUJ_10428_cut3     DANQKCPDAVLIAGGYSQGAALAAASVTDVDASIREKIAGVVLFGYTKNLQNRGKIPSYP      180
Cut5_#62            DANQKCPDAVLIAGGYSQGAALAAASVTDVDAGIREKIAGVVLFGYTKNLQNRGKIPSYP      180
FOZG_16529_cut3     DANQKCPDAVLIAGGYSQGAALAAASVTDVDAGIREKIAGVVLFGYTKNLQNRGKIPSYP      180
Cut5_#38            DANQKCPDAVLIAGGYSQGAALAAASVTDVDAGIREKIAGVVLFGYTKNLQNRGKIPSYP      180
FoCut5              DANQKCPDAVLIAGGYSQGAALAAASVTDVDAGIREKIAGVVLFGYTKNLQNRGKIPSYP      164
                    ********************************.***************************

FFUJ_10428_cut3     EDRTKVFCNTGDLVCTGSLIVAAPHLAYQSDASNGAPEFLIQKADAAGAA  230
Cut5_#62            EDRTKVFCNTGDLVCTGSLIVAAPHLAYQSAASGAAPEFLIQKADAAGAA  230
FOZG_16529_cut3     EDRTKVFCNTGDLVCTGSLIVAAPHLAYQSAASGAAPEFLIQKADAAGAA  230
Cut5_#38            EDRTKVFCNTGDLVCTGSLIVAAPHLAYQSAASGAAPEFLIQKADAAGAA  230
FoCut5              EDRTKVFCNTGDLVCTGSLIVAAPHLAYQSAASGAAPEFLIQKADAAGAA  214
                    ****************************** **..***************
```

Alignment Cut1 and the reference Cutinases from NCBI

```
cut1_#62            MKFTAVLSFFAAASVALPTSNPSSELE------------VRQAGSITRDDLSNGASSACP      48
FFUJ_06741_cut1     MKFTTILSLFAATAVALPTSNPLSEREV------LAELEKRQSGSITRDDLSNGASSACP      54
cut1_#38            MKFTAVLSFFAAASVALPTSNPSSELEVRAELEVRAELEERQSGSITRDDLSNGASSACP      60
FOZG_16527_cut1     MKFIAVLSFFAAASVALPTSNPSSELEV------RAELEERQSGSITRDDLSNGASSACP      54
                    *** ::**:***::******** ** *             **:*****************

cut1_#62            PVIFIYARGSTELGNLGTLGPRVASVLESYYGNNKVWIQGVGGAYRATLGDNALPRGTSA      108
FFUJ_06741_cut1     PVIFIYARGSTELGNLGTLGPRVASVLESNYGSNGVWIQGVGGAYRATLGDNALPRGTSS      114
cut1_#38            PVIFIYARGSTELGNLGTLGPRVASVLESNYGSNGVWIQGVGGAYRATLGDNALPRGTSS      120
FOZG_16527_cut1     PVIFIYARGSTELGNLGTLGPRVASVLESNYGSNGVWIQGVGGAYRATLGDNALPRGTSS      114
                    **************************** **.* *************************:

cut1_#62            AAIREMIGLFNLASTKCPSAKIVAGGYSQGAALAAASIEDLSTTVRNKVVGTVLFGYTKN      168
FFUJ_06741_cut1     AAIREMIGLFNLANSKCPSAKIVAGGYSQGAALAAASIEDLSTSVRNKVVGTVLFGYTKN      174
cut1_#38            AAIREMIGLFNLANSKCPSAKIVAGGYSQGAALAAASIEDLSTAVRNKVVGTVLFGYTKN      180
FOZG_16527_cut1     AAIREMIGLFNLANSKCPSAKIVAGGYSQGAALAAASIEDLSTAVRNKVVGTVLFGYTKN      174
                    *************.:****************************:****************

cut1_#62            LQNLGRIPNYPRERTLVFCNVGDLVCTGSLIVAAPHLAYQSDASGPAPQFLIQRVAATSI      228
FFUJ_06741_cut1     LQNLGRIPNYPRERTLVFCNFGDLVCTGSLIVAAPHLAYQSDASGPAPQFLIQRVAATSI      234
cut1_#38            LQNLGRIPNYPRERTLVFCNIGDLVCTGSLIVAAPHLAYQSDASGPAPQFLIQRVAATSI      240
FOZG_16527_cut1     LQNLGRIPNYPRERTLVFCNIGDLVCTGSLIVAAPHLAYQSDASGPAPQFLIQRVAATSI      234
```

Supplementary data 2	Statistics Figure 2d				
Table Analyzed	%impranil present spend medium column				
Two-way RM ANOVA	Matching: Stacked				
Assume sphericity?	No				
Alpha	0,05				
Source of Variation	% of total variation	P value	P value summary	Significant?	Geisser-Greenhouse's epsilon
Time x Column Factor	9,777	<0,0001	****	Yes	
Time	3,306	<0,0001	****	Yes	0,436
Column Factor	86,41	<0,0001	****	Yes	
Subject	0,2724	0,0002	***	Yes	
ANOVA table	SS	DF	MS	F (DFn, DFd)	P value
Time x Column Factor	0,2418	16	0,01512	F (16, 40) = 106,0	P<0,0001
Time	0,08178	4	0,02044	F (1,744, 17,44) = 143,4	P<0,0001
Column Factor	2,137	4	0,5344	F (4, 10) = 793,1	P<0,0001
Subject	0,006737	10	0,0006737	F (10, 40) = 4,725	P=0,0002
Residual	0,005704	40	0,0001426		
Data summary					
Number of columns (Column Factor)	5				
Number of rows (Time)	5				
Number of subjects (Subject)	15				

Number of missing values	0

Within each row, compare columns (simple effects within rows)

Number of families	5
Number of comparisons per family	4
Alpha	0,05

Dunnett's multiple comparisons test	Mean Diff,	95,00% CI of diff,	Below threshold?	Summary	Adjusted P Value
0					
control vs. 62Cut1	0,0192	-0,01765 to 0,05605	No	ns	0,2633
control vs. 62Cut3	0,1343	0,05921 to 0,2095	Yes	*	0,0101
control vs. 38Cut1	0,06597	-0,04329 to 0,1752	No	ns	0,1519
control vs. 38Cut3	0,1905	0,1326 to 0,2483	Yes	**	0,0011
1					
control vs. 62Cut1	0,0017	-0,004561 to 0,007961	No	ns	0,5794
control vs. 62Cut3	0,2637	0,1137 to 0,4136	Yes	*	0,0166
control vs. 38Cut1	0,002967	-0,03189 to 0,03782	No	ns	0,9505
control vs. 38Cut3	0,4383	0,4255 to 0,4512	Yes	****	<0,0001

2					
control vs. 62Cut1	-0,002967	-0,009964 to 0,004030	No	ns	0,4101
control vs. 62Cut3	0,3724	0,2357 to 0,5091	Yes	**	0,0069
control vs. 38Cut1	-0,008267	-0,03911 to 0,02258	No	ns	0,494
control vs. 38Cut3	0,4308	0,4233 to 0,4383	Yes	****	<0,0001
3					
control vs. 62Cut1	-0,0044	-0,01002 to 0,001217	No	ns	0,1035
control vs. 62Cut3	0,4025	0,3438 to 0,4611	Yes	***	0,001
control vs. 38Cut1	-0,0059	-0,04566 to 0,03386	No	ns	0,7857
control vs. 38Cut3	0,4112	0,4047 to 0,4178	Yes	****	<0,0001
4					
control vs. 62Cut1	-0,005167	-0,01142 to 0,001091	No	ns	0,0825
control vs. 62Cut3	0,4121	0,3860 to 0,4383	Yes	****	<0,0001
control vs. 38Cut1	-0,0062	-0,04509 to 0,03269	No	ns	0,7608
control vs. 38Cut3	0,4067	0,3999 to 0,4134	Yes	****	<0,0001

Test details	Mean 1	Mean 2	Mean Diff,	SE of diff,	N1	N2	q	DF
0								
control vs. 62Cut1	0,5511	0,5319	0,0192	0,009075	3	3	2,116	3,583
control vs. 62Cut3	0,5511	0,4168	0,1343	0,01642	3	3	8,181	2,966
control vs. 38Cut1	0,5511	0,4852	0,06597	0,02124	3	3	3,106	2,547
control vs. 38Cut3	0,5511	0,3607	0,1905	0,01382	3	3	13,79	3,397
1								
control vs. 62Cut1	0,5489	0,5472	0,0017	0,001293	3	3	1,315	2,746
control vs. 62Cut3	0,5489	0,2853	0,2637	0,02315	3	3	11,39	2,01
control vs. 38Cut1	0,5489	0,546	0,002967	0,005841	3	3	0,5079	2,171
control vs. 38Cut3	0,5489	0,1106	0,4383	0,002744	3	3	159,7	2,869
2								
control vs. 62Cut1	0,5455	0,5485	-0,002967	0,001816	3	3	1,634	3,956
control vs. 62Cut3	0,5455	0,1731	0,3724	0,02115	3	3	17,61	2,013
control vs. 38Cut1	0,5455	0,5538	-0,008267	0,005291	3	3	1,562	2,222
control vs. 38Cut3	0,5455	0,1147	0,4308	0,001915	3	3	224,9	3,852
3								
control vs. 62Cut1	0,5439	0,5483	-0,0044	0,001451	3	3	3,032	3,919
control vs. 62Cut3	0,5439	0,1414	0,4025	0,009288	3	3	43,33	2,057
control vs. 38Cut1	0,5439	0,5498	-0,0059	0,006494	3	3	0,9086	2,118

control vs. 38Cut3	0,5439	0,1326	0,4112	0,001128	3	3	364,6	2,224
4								
control vs. 62Cut1	0,5433	0,5484	-0,005167	0,001404	3	3	3,679	3,082
control vs. 62Cut3	0,5433	0,1311	0,4121	0,004643	3	3	88,76	2,303
control vs. 38Cut1	0,5433	0,5495	-0,0062	0,006458	3	3	0,96	2,152
control vs. 38Cut3	0,5433	0,1366	0,4067	0,001757	3	3	231,4	3,999

Statistics Figure 3d

Table Analyzed	4h Impranil column
Two-way RM ANOVA	Matching: Stacked
Assume sphericity?	No
Alpha	0,05

Source of Variation	% of total variation	P value	P value summary	Significant?	Geisser-Greenhouse's epsilon
Time x Column Factor	17,1	<0,0001	****	Yes	
Time	39,86	<0,0001	****	Yes	0,6686
Column Factor	42,62	<0,0001	****	Yes	
Subject	0,2703	<0,0001	****	Yes	

ANOVA table	SS	DF	MS	F (DFn, DFd)	P value
Time x Column Factor	0,2134	16	0,01334	F (16, 40) = 297,9	P<0,0001
Time	0,4975	4	0,1244	F (2,674, 26,74) = 2778	P<0,0001
Column Factor	0,5319	4	0,133	F (4, 10) = 394,2	P<0,0001
Subject	0,003373	10	0,0003373	F (10, 40) = 7,533	P<0,0001
Residual	0,001791	40	0,00004478		

Data summary	
Number of columns (Column Factor)	5
Number of rows (Time)	5
Number of subjects (Subject)	15
Number of missing values	0
Within each row, compare columns (simple effects within rows)	
Number of families	5
Number of comparisons per family	4
Alpha	0,05

Dunnett's multiple comparisons test	Mean Diff,	95,00% CI of diff,	Below threshold?	Summary	Adjusted P Value
0					
contrl vs. Cut	0,004667	-0,02473 to 0,03407	No	ns	0,9091
contrl vs. LCC	-0,003233	-0,03897 to 0,03250	No	ns	0,9807
contrl vs. 63	0,01527	-0,006501 to 0,03703	No	ns	0,1394
contrl vs. 38	0,03013	-0,008683 to 0,06895	No	ns	0,0968
1					
contrl vs. Cut	0,02727	-0,01049 to 0,06503	No	ns	0,128
contrl vs. LCC	0,02477	-0,01248 to 0,06202	No	ns	0,147
contrl vs. 63	0,1189	0,08132 to 0,1565	Yes	***	0,0009
contrl vs. 38	0,1458	0,1066 to 0,1851	Yes	**	0,0015

2						
contrl vs. Cut	0,04483	0,01822 to 0,07145	Yes	**		0,0083
contrl vs. LCC	0,09123	0,06558 to 0,1169	Yes	***		0,0005
contrl vs. 63	0,1928	0,1641 to 0,2214	Yes	***		0,0009
contrl vs. 38	0,253	0,2295 to 0,2765	Yes	****	<0,0001	
3						
contrl vs. Cut	0,0932	0,07099 to 0,1154	Yes	***		0,0003
contrl vs. LCC	0,1939	0,1482 to 0,2397	Yes	**		0,0012
contrl vs. 63	0,2419	0,2192 to 0,2647	Yes	***		0,0005
contrl vs. 38	0,3943	0,3643 to 0,4244	Yes	****	<0,0001	
4						
contrl vs. Cut	0,1933	0,09895 to 0,2877	Yes	*		0,0111
contrl vs. LCC	0,3148	0,2812 to 0,3484	Yes	***		0,0001
contrl vs. 63	0,2957	0,2806 to 0,3108	Yes	****	<0,0001	
contrl vs. 38	0,4102	0,3940 to 0,4265	Yes	****	<0,0001	

Test details	Mean 1	Mean 2	Mean Diff,	SE of diff,	N1	N2	q	DF
0								
contrl vs. Cut	0,5404	0,5357	0,004667	0,007294	3	3	0,6398	3,632
contrl vs. LCC	0,5404	0,5436	-0,003233	0,00832	3	3	0,3886	3,261
contrl vs. 63	0,5404	0,5251	0,01527	0,005619	3	3	2,717	3,914
contrl vs. 38	0,5404	0,5103	0,03013	0,008787	3	3	3,429	3,122
1								
contrl vs. Cut	0,5167	0,4895	0,02727	0,009654	3	3	2,824	3,841
contrl vs. LCC	0,5167	0,492	0,02477	0,008847	3	3	2,799	3,368
contrl vs. 63	0,5167	0,3978	0,1189	0,009557	3	3	12,44	3,802
contrl vs. 38	0,5167	0,3709	0,1458	0,008235	3	3	17,71	2,801
2								
contrl vs. Cut	0,5243	0,4794	0,04483	0,00691	3	3	6,488	3,959
contrl vs. LCC	0,5243	0,433	0,09123	0,006689	3	3	13,64	3,993
contrl vs. 63	0,5243	0,3315	0,1928	0,004705	3	3	40,97	2,129
contrl vs. 38	0,5243	0,2713	0,253	0,006034	3	3	41,92	3,877
3								
contrl vs. Cut	0,5311	0,4379	0,0932	0,005648	3	3	16,5	3,803
contrl vs. LCC	0,5311	0,3371	0,1939	0,009212	3	3	21,05	2,66
contrl vs. 63	0,5311	0,2891	0,2419	0,003514	3	3	68,85	2,009
contrl vs. 38	0,5311	0,1367	0,3943	0,00695	3	3	56,74	3,226

4

contrl vs. Cut	0,5405	0,3472	0,1933	0,0157	3	3	12,31	2,156
contrl vs. LCC	0,5405	0,2257	0,3148	0,007134	3	3	44,12	2,849
contrl vs. 63	0,5405	0,2447	0,2957	0,003711	3	3	79,7	3,572
contrl vs. 38	0,5405	0,1302	0,4102	0,003305	3	3	124,1	2,692

Statistics figure 3e

Table Analyzed	BHET column				
Two-way RM ANOVA	Matching: Stacked				
Assume sphericity?	Yes				
Alpha	0,05				
Source of Variation	% of total variation	P value	P value summary	Significant?	
Time x Enzyme	0,8579	0,0022	**	Yes	
Time	1,848	<0,0001	****	Yes	
Enzyme	95,69	<0,0001	****	Yes	
Subject	1,421	0,0046	**	Yes	
ANOVA table	SS	DF	MS	F (DFn, DFd)	P value
Time x Enzyme	0,002707	3	0,0009024	F (3, 8) = 12,42	P=0,0022
Time	0,005831	1	0,005831	F (1, 8) = 80,23	P<0,0001
Enzyme	0,302	3	0,1007	F (3, 8) = 179,5	P<0,0001
Subject	0,004485	8	0,0005606	F (8, 8) = 7,713	P=0,0046
Residual	0,0005815	8	0,00007268		
Difference between row means					
Mean of 24	0,2341				
Mean of 48	0,2029				
Difference between means	0,03118				
SE of difference	0,003481				
95% CI of difference	0,02315 to 0,03920				
Data summary					
Number of columns (Enzyme)	4				
Number of rows (Time)	2				
Number of subjects (Subject)	12				

Number of missing values	0

Compare column means (main column effect)

Number of families	1
Number of comparisons per famil	3
Alpha	0,05

Šídák's multiple comparisons test	Mean Diff,	95,00% CI	Below threshold?	Summary	Adjusted P Value
Control vs. PET	0,2663	0,2252 to (	Yes	****	<0,0001
Control vs. 62Cut3	0,009333	-0,03174 tc	No	ns	0,8852
Control vs. 38Cut3	0,1707	0,1297 to (	Yes	****	<0,0001

Test details	Mean 1	Mean 2	Mean Diff,	SE of diff,	N1	N2	t	DF
Control vs. PET	0,3301	0,06383	0,2663	0,01367	6	6	19,48	8
Control vs. 62Cut3	0,3301	0,3208	0,009333	0,01367	6	6	0,6827	8
Control vs. 38Cut3	0,3301	0,1594	0,1707	0,01367	6	6	12,49	8

Supplemental Data 3 – absolute values pNp assays

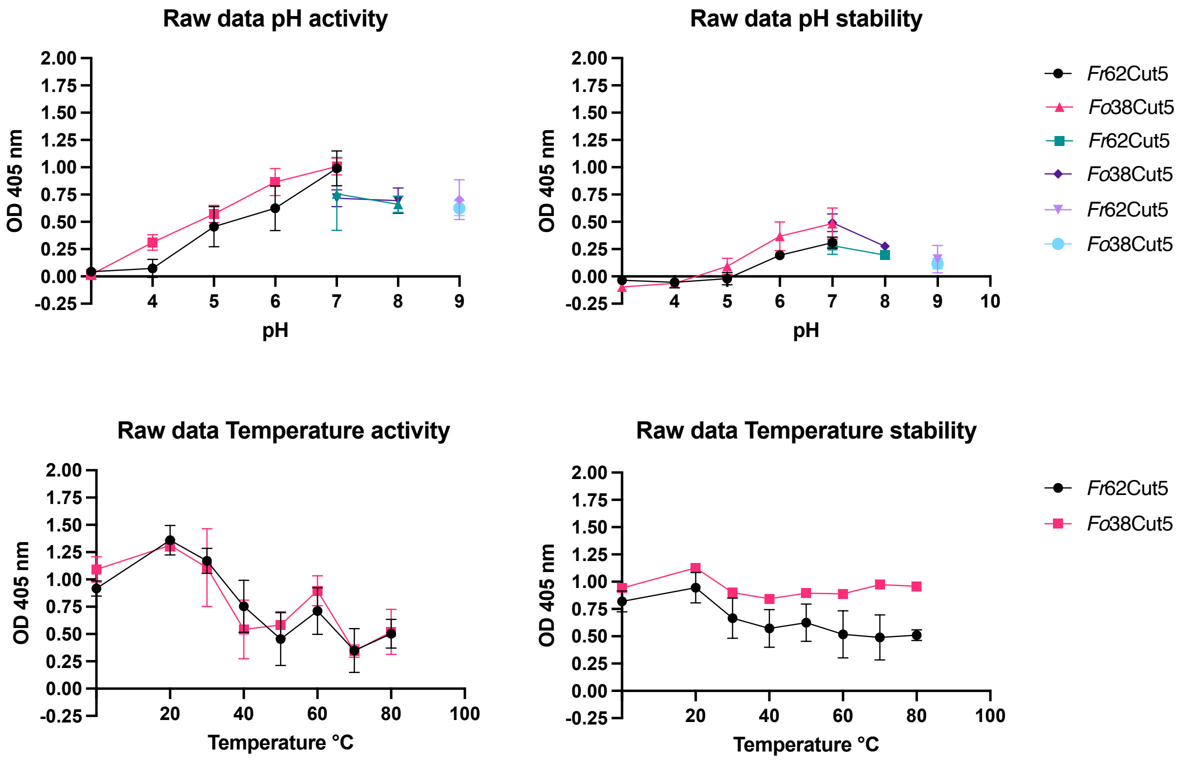

Supplemental data 3: The corrected absolute values of optimal conditions determined using para nitrophenyl dodecanoate. The top graphs display optimal pH and pH stability the black (*Fr*62Cut5) and magenta line (*Fo*38Cut5) represent the assays performed in citrate buffer, the purple (Fr62Cut5) and turquoise line (*Fo*38Cut5) represent Tris-HCl buffer and the lilac (*Fr*62Cut5) and light blue line (*Fo*38Cut5) represent glycin buffer. The bottom figures display the absolute values of the temperature activity and stability the black line represents *Fr*62Cut5 and the magenta line represents *Fo*38Cut5. All assays were performed in triplicate, the error bars represent the standard deviation.

Supplemental data 4 – optimal conditions Impranil degradation

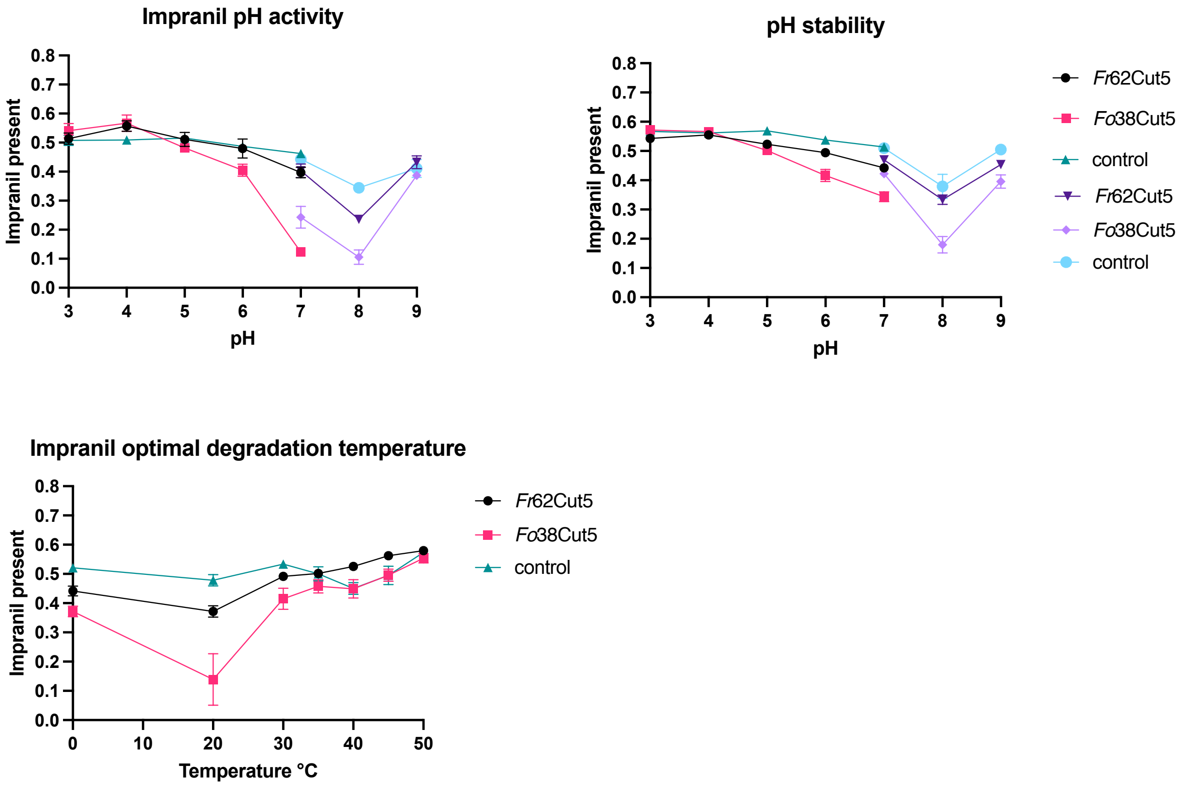

Supplemental data 4: The optimal conditions determined using Impranil-DLN.

The top graphs display optimal pH and pH stability the black (Fr62Cut5), magenta line (Fo38Cut5) and turquoise line (control) represent the assays performed in citrate buffer. The purple (Fr62Cut5), lilac (Fo38Cut5) and light blue line (control) represent Tris-HCL (pH 7 and 8) and glycin (pH 9) The bottom figure displays the optimal temperature activity the black line represents Fr62Cut5, and the magenta line represents Fo38Cut5. All assays were performed in triplicate, the error bars represent the standard deviation.

Supplementary table 1. Amino acid sequences used for phylogenetic analysis

	enzyme	species	Accession code	Reference	plastic/function
1	*An* Cut1	*Aspergillus nidulans*	Q5B2C1	(Bermúdez-García et al., 2019)	cutin degradation
2	*An* Cut2	*Aspergillus nidulans*	Q5AVY9	(Bermúdez-García et al., 2019; Castro-Ochoa et al., 2012)	cutin degradation
3	*An* Cut3	*Aspergillus nidulans*	Q5AX00	(Bermúdez-García et al., 2019)	cutin degradation
4	*An* Cut4	*Aspergillus nidulans*	C8VJF5	(Bermúdez-García et al., 2019)	cutin degradation
5	*Ang* Cut1	*Aspergillus niger*	Supplemental data	(Altammar et al., 2022)	
6	*Ang* Cut2	*Aspergillus niger*	Supplemental data	(Altammar et al., 2022)	
7	*Ang* Cut3	*Aspergillus niger*	Supplemental data	(Altammar et al., 2022)	PET/PCL

8	*Ang* Cut4	*Aspergillus niger*	Supplemental data	(Altammar et al., 2022)	
9	*Ang* Cut5	*Aspergillus niger*	Supplemental data	(Altammar et al., 2022)	
10	*Ao* Ami	*Aspergillus oryzae*	Q12559	(Masaki et al., 2020)	PUR
11	*Ao* CutL1	*Aspergillus oryzae*	P52956	(Maeda et al., 2005; Oda et al., 2000)	PLA
12	*Fo* Cut5	*Fusarium oxysporum*	X0BTD8	(Dimarogona et al., 2015)	PET
13	*Fs* C	*Fusarium solani pisi*	AAA33335.1	(Silva et al., 2005)	PET
14	*Fs* CutA	*Fusarium solani*	Q99174	(Crowhurst et al., 1997)	virulence

15	*Fv* Cut1	*Fusarium vanetteniii*	P00590	(Martinez et al., 1992)	PET
16	*Fv* Cut2	*Fusarium vanetteniii*	Q96UT0	(Li et al., 2002)	virulence
17	*Fv* Cut3	*Fusarium vanetteniii*	Q96US9	(Li et al., 2002)	virulence
18	*Hi* C	*Humicola insolens*	A0A075B5G4	(Brackmann et al., 2023; Carniel et al., 2017; Ronkvist et al., 2009)	PET
19	LCC	Compost	G9BY57	(Sulaiman et al., 2012)	PET
20	*Tf* Cut1	*Thermobifida fusca*	E5BBQ2		PET
21	*Tf* Cut2	*Thermobifida fusca*	E5BBQ3_THEFU	(Roth et al., 2014)	PET

enzyme	AA
An Cut1	NPIRLDQRQITGNELRDGSCHDVTFIFARGSTELGYLGSTVGPATCNVLKLRKPGQVACQGVAPAYIADLASNFLPQGTN<br>QIAINEAKSLFELAASKCPNTKIVAGGYSQGAAVMHAAISTLSSTVQDQIKGVVLFGDTRNKQDGGRIPNFPTDKTKIICA<br>FGDLVCEGTLVITAAHLSYIDDVPDAADFLVGKL
An Cut2	SPLNLDERQLGSSSGNDLRDGDCKPVTFIFARASTEPGLLGMSTGPAVCNDLKADASLGGVACQGVGPKYTAGLAENA<br>LPQGTSSAAINEAKELFELAASKCPDTRIVAGGYSQGTAVMHGAIPDLSDEIKDKIAGVVLFGDTRNKQDGGQIKNFPKD<br>KIKIYCATGDLVCDGTLVVTAAHFTYVANTGEASKWLEQQLASMPASTSTSSSSSSSSSAPASQTSQSSGLSSWFSGLGN
An Cut3	TPLPSDTDVSLERRQSMNSNDLEKGDCKSVAFIFARGSTEIGNMGFVVGPGVCSNLKSTLGSDKVACQGVGGAYTAGLI<br>QNALPANTDSGSIKEAVKMFDLAAKCPDTQIVAGGYSQGSAVIDNAIQKLDDSTRDRVKGVVLFGFTRNLQDKGQIPGY<br>PKDQTKVYCAVGDLVCSGTLIITASHMTYGLNAGDAAKFLASQVSV
An Cut4	TGVAYSARQVTPTAPLPRLRGSSTSNDVTDNSGCKELTFIFARGTTEIGNMGTVVGPKVGEALKSLTGNKAAIQGVDYP<br>ADAAGNAALGGSGGPKMASLVETALKQCPDTKIVLGGYSQGAMVVHNAASKLSSGQVVGAVTFGDPFKSQKPDNIDQ<br>FKTFCASGDPVCLNGANVMAHLSYGNDAQTAAQFLVSAAGL
Ang Cut1	MAPLKSLLLGASLATLALSTPLATDAENLYARQFGTGSTANELEQGSCKDVTLIFARGSTELGNMGTVIGPPLCDNLKSK<br>LGSDKVACQGVGGQYSAGLVQNALPQNTDPGSISAAKQMFEEANSKCPNTKIVAGGYSQGSAVIDNAVQELSTTVKDQ<br>VKGVVLFGFTRNVQDHGQIPNYPKDDVKVYCAVGDLVCDDTLVVTAMHLTYGMDAGDAASFLAEKVQSSSSSTTSSS<br>SDAASSSSAAGTSSSGLSGLSSFFGGL
Ang Cut2	MKLPYFLLGLAGLAAASPMGLAERQLSDGNELREGSCKPIIFIFARASTEPGLLGISTGPAVCNDLKMAKAGQVLCQGVG<br>PAYTADLMSNALPDNTSPAAISESESLFKLAASKCPNSQILAGGYSQGTAVMDDSIKQLPDDVKDKIKGVVLFGYTRNA<br>QEGGQIGNFPKDKVKIYCAMGDLVCDGTLIVTAAHFTYVMNTGEASQWLESKLSDTTTSSLTGSSSSDTSSSTSTGDSSS<br>ESSSAAGLGGLSGLTGLGSSTSGGFPSLASLF
Ang Cut3	MKSFITLSLLATALGAPTATHQARQFDLSSLTSGLSALTSGSAASSGSTGSTGSETTTGSSGLSALASLFPSSSTSSTTGSTS<br>GSGSSSSSSDGLSSLMSMFSSSGSGSSTENGVTQHSGSCKKLTFIFARGTTEIGNMGTVVGPEVASELATLTGNQVTVQG<br>VNYPADWEGNVSLGSSGGPTMASYVKEALQQCPNTKVVLGGYSQGSMVVHYAANQLSADQLSGAVLFGDPLKMEGV<br>GKLSSSKVKEFCASGDPVCENGMNVMAHLTYGSDAKEAAQFLVQAAGVSSS

Ang Cut4	MLSRIVTTLAFNALLGFSRATLLNSTDCADIHFMLARGTTEDYPGTTYSMAELVAENTTLSTNYENIIYPAVSETESDSYFI<br>GRAAVGSQVNRYAADCPNSRIVLISYSQGAMIVGDALAGGGGDSTLGNATQPLVSEDVSKHIAANVYYGNPRHAPYQP<br>YNMGNNTWNVTGKYPRLDYQINYLHDRYRHVTADWCNDGDGVCSPSEGADALSLHMAYANDYDPIAAAWILEKLR<br>A
Ang Cut5	MFKNIATAALLSSALASASTAHTNGKAPCAKVHMMLARGTTESYPGLLGSLTELVMDAVPDSDYENIIYPATQEGSTPS<br>YEEGIYNGTAQLKAYVKACPESKVVLFGYSQGAMVVSDMLAGGGDNGTLGNITAPAVDPETGSHIAAVLLYGDPRHM<br>PNQTYNVGDVTATGKYPRTPEQLAALSQYADRLHDYCDNKDGVCDAAGTNLSAHLAYATIWDKVAATWVESMMQK
Ao Ami	MPSASWEDLAADKRARLEKSIPDEWKFKSVPIEGSVIDLPEKSGILSPSEIKITNSSATELVAQLANGTLKSVDVTLAFCKR<br>AALAHQLVNCAHDFFPELALAQARELDRYFETHKKPVGPLHGLPISLKDQLRVKGTETCMAYISWLGKRDTSDSILTAL<br>LRKAGAVFLVKTSVPQTLMVCETVNNIIGRTSNPRNLNLSCGGSSGGEGAMIAMRGGAIGIGTDIGGSIRVPAAFNSLYGI<br>RPSHGRLPYGGMTNSMEGQETIHSVVGPIAHSAQDVRLFLQSVLKEEPWKYDSKVIPLPWREAEENAAQAKIAEKSLNF<br>AFYDFDGVVRPHPPITRGVEIVRSTLEKDGHTVAPWTPYKHAFAVDLANKIYAADGSTDVYKHINASGEPAIPNIKDLM<br>NPNLPKADLNEVWDAQLQKWRYQCEYLDKWREWEERTGKELDAIIAPVAATAAVRHNQFRYYGYATVFNVLDYTSV<br>VVPVTYADKAVDHRLADYQPVSDMDKAVYAEYDPEVYHGAPVAVQIIGRRLSEERTLAIAEYVGKLLGH
Ao CutL1	SPVDLQDRQLTGGDELRDGPCKPITFIFARASTEPGLLGISTGPAVCNRLKLARSGDVACQGVGPRYTADLPSNALPEGT<br>SQAAIAEAQGLFEQAVSKCPDTQIVAGGYSQGTAVMNGAIKRLSADVQDKIKGVVLFGYTRNAQERGQIANFPKDKVK<br>VYCAVGDLVCLGTLIVAPPHFSYLSDTGDASDFLLSQLG
Fo Cut5	LPAGQDAAALEARQLGGSITRNDLANGNSGSCPGVIFIYARGSTESGNLGTLGPRVASKLEAKYGKNGVWIQGVGGAY<br>RATLGDNALPRGTSSAAIREMLGHFSDANQKCPDAVLIAGGYSQGAALAAASVTDVDAGIREKIAGVVLFGYTKNLQN<br>RGKIPSYPEDRTKVFCNTGDLVCTGSLIVAAPHLAYQSAASGAAPEFLIQKADAAGAA
Fs C	LPTSNPAQELEARQLGRTTRDDLINGNSASCADVIFIYARGSTETGNLGTLGPSIASNLESAFGKDGVWIQGVGGAYAAT<br>LGDNALPRGTSSAAIREMLGLFQQANTKCPDATLIAGGYSQGAALAAASIEDLDSAIRDKIAGTVLFGYTKNLQNRGRIP<br>NYPADRTKVFCNTGDLVCTGSLIVAAPHLAYGPDARGPAPEFLIEKVRAVRGSA
Fs CutA	LPTSNPAQELEARQLGRTTRDDLINGNSASCADVIFIYARGSTETGNLGTLGPSIASNLESAFGTDGVWIQGVGGAYRAT<br>LGDNALPRGTSSAAIREMLGLFQQANTKCPDATLIAGGYSQGAALAAASIEDLDSAIRDKIAGTVLFGYTKNLQNRGRIP<br>NYPADRTKVFCNVGDLVCTGSLIVAAPHLAYGPDARGPAPEFLIEKVRAVRGSA

Fv Cut1	GRTTRDDLINGNSASCRDVIFIYARGSTETGNLGTLGPSIASNLESAFGKDGVWIQGVGGAYRATLGDNALPRGTSSAAI<br>REMLGLFQQANTKCPDATLIAGGYSQGAALAAASIEDLDSAIRDKIAGTVLFGYTKNLQNRGRIPNYPADRTKVFCNTG<br>DLVCTGSLIVAAPHLAYGPDARGPAPEFLIEKVRAVRGSA
Fv Cut2	LPTSHPVQELEARQLGGGTTRNDLTNGNSASCADVIFIYARGSTETGNLGTLGPSIASKLESAFGRDGVWIQGVGGAYRA<br>TLGDNSLPRGTSSAAIREMLGLFQQANTKCPDATLIAGGYSQGAALGAASVEDLDSAIRDKIAGTVLFGYTKNLQNHGRI<br>PNFPADRTKVFCNTGDLVCTGSLIIAAPHLTYGPDARGPAPEFLIEKVRAVRGSA
Fv Cut3	LPTSHPVQELEARQLGGGTTRNDLTNGNSASCADVIFIYARGSTETGNLGTLGPSIASKLESAFGRDGVWIQGVGGAYRA<br>TLGDNSLPRGTSSAAIREMLGLFQQPNTKCPDATLIAGGYSQGAALAAASVEDLDSAIRDKIAGTVLFGYTKNLQNHGRI<br>PNFPADRTKVFCNTGDLVCTGSLIIAAPHLTYGPDARGPAPEFLIEKVRAVRGSA
Hi C	QLGAIENGLESGSANACPDAILIFARGSTEPGNMGITVGPALANGLESHIRNIWIQGVGGPYDAALATNFLPRGTSQANID<br>EGKRLFALANQKCPNTPVVAGGYSQGAALIAAAVSELSGAVKEQVKGVALFGYTQNLQNRGGIPNYPRERTKVFCNVG<br>DAVCTGTLIITPAHLSYTIEARGEAARFLRDRIRA
LCC	QSNPYQRGPNPTRSALTADGPFSVATYTVSRLSVSGFGGGVIYYPTGTSLTFGGIAMSPGYTADASSLAWLGRRLASHGF<br>VVLVINTNSRFDYPDSRASQLSAALNYLRTSSPSAVRARLDANRLAVAGHSMGGGGTLRIAEQNPSLKAAVPLTPWHTD<br>KTFNTSVPVLIVGAEADTVAPVSQHAIPFYQNLPSTTPKVYVELDNASHFAPNSNNAAISVYTISWMKLWVDNDTRYRQ<br>FLCNVNDPALSDFRTNNRHCQ
Tf Cut1	ANPYERGPNPTDALLEARSGPFSVSEENVSRLGASGFGGGTIYYPRENNTYGAVAISPGYTGTQASVAWLGKRIASHGF<br>VVITIDTITTLDQPDSRARQLNAALDYMINDASSAVRSRIDSSRLAVMGHSMGGGGSLRLASQRPDLKAAIPLTPWHLNK<br>NWSSVRVPTLIIGADLDTIAPVLTHARPFYNSLPTSISKAYLELDGATHFAPNIPNKIIGKYSVAWLKRFVDNDTRYTQFLC<br>PGPRDGLFGEVEEYRSTCPF
Tf Cut2	ANPYERGPNPTDALLEASSGPFSVSEENVSRLSASGFGGGTIYYPRENNTYGAVAISPGYTGTEASIAWLGERIASHGFVV<br>ITIDTITTLDQPDSRAEQLNAALNHMINRASSTVRSRIDSSRLAVMGHSMGGGGTLRLASQRPDLKAAIPLTPWHLNKNW<br>SSVTVPTLIIGADLDTIAPVATHAKPFYNSLPSSISKAYLELDGATHFAPNIPNKIIGKYSVAWLKRFVDNDTRYTQFLCPG<br>PRDGLFGEVEEYRSTCPF

Supplementary Table 2: **BHET conversion rate enzymes based on 24h measurement**

µmol/h/mg enzyme	24h			average
PET	5,07	5,86	5,98	5,63
62Cut5	-0,26	0,63	0,40	0,26
38Cut5	3,33	3,28	2,60	3,07

Supplementary table 3. **Strains used in this study**

Genus	Strain name	Genotype	Reference
Fusarium	FORL	wild-type; *F. oxysporum. f. sp. Radicis lycopersici*	De Weert et al., 2004
	Fusarium # 38	fungal endophytic isolate # 38	This study
	Fusarium # 60	fungal endophytic isolate # 60	This study
	Fusarium # 61	fungal endophytic isolate # 61	This study
	Fusarium # 62	fungal endophytic isolate # 62	This study
Aspergillus niger	N400	wild-type NRRL3; ATCC9092; CBS120.49	Bos et al., 1988
	MA1048.1	*kusA:: DR-amdS-DR, ΔglaA::glaA_LS-K1, ΔaamA::glaA_LS-K1, ΔagdA-amyA-prtT::glaA_LS-K1, ΔpepA::glaA_LS-K4, ΔpepB::glaA_LS-K4, ΔpepN::glaA_LS-K5, ΔNRRL3_10267::glaA_LS-K1+K2, ΔgoxC::glaA_LS-K1+K3, ΔoahA::glaA_LS-K1+K3, ΔNRRL3_06629::glaA_LS-K1+K2, constitutive mutation in amyR*	Arentshorst et al., 2023
	MA1084.2	*ΔNRRL3_10267::PglaA-62-cut1-HIS-TglaA ΔgoxC::PglaA-62-cut1-HIS-TglaA ΔoahA::PglaA-62-cut1-HIS-TglaA* in MA1048.1	This study
	MA1085.2	*ΔNRRL3_10267::PglaA-62-cut5-HIS-TglaA ΔgoxC::PglaA-62-cut5-HIS-TglaA ΔoahA::PglaA-62-cut5-HIS-TglaA ΔNRRL3_06629::PglaA-62-cut5-HIS-TglaA* in MA1048.1	This study
	MA1086.6	*ΔNRRL3_10267::PglaA-38-cut1-HIS-TglaA ΔgoxC::PglaA-38-cut1-HIS-TglaA ΔoahA::PglaA-38-cut1-HIS-TglaA ΔNRRL3_06629::PglaA-38-cut1-HIS-TglaA* in MA1048.1	This study
	MA1089.3	*ΔNRRL3_10267::PglaA-38-cut5-HIS-TglaA ΔoahA::PglaA-38-cut5-HIS-TglaA ΔNRRL3_06629::PglaA-38-cut5-HIS-TglaA* in MA1048.1	This study

Supplemental Table 4. **Overview of the primers used in this study**

**Oligo**	**Sequence 5'-3'**	**Used for**	**Fragment size**		**Strain**	**Ref**
			**PCR**	**Fusion PCR**		
**Internal Transcribed Spacer (ITS) sequencing**						
V9g	TTACGTCCCTGCCCTTTGTA	Sequencing ITS *Fusarium* strains 62 and 38	680 bp			G S de Hoog and A H Gerrits van den Ende, 1998
ITS4	TCCTCCGCTTATTGATATGC	Sequencing ITS *Fusarium* strains 62 and 38				White et al., 1990
**Amplification of cut1 and cut5 from Fusarium strains 62 and 38**						
Fus_cut1_P1f	ATGAAGTTCAYCRCYRTCCTCTC	cut1 ORF *Fusarium* strains 62 and 38	789 bp (62-cut1)			
Fus_cut1_P2r	CTAGATAGAGGTAGCAGCAA C	cut1 ORF *Fusarium* strains 62 and 38	826 bp (38-cut1)			
Fus_cut5_P1f	ATGAAGTTCTCCATCATCTCTAC	cut5 ORF Fusarium strains 62 and 38	750 bp (62-cut5)			
Fus_cut5_P2r	TYARGCRGCTCCAGCAGCATCAG	cut5 ORF Fusarium strains 62 and 38	758 bp (38-cut5)			

**Construction of *PglaA-cut-HIS-TglaA* Donor DNA**						
underlined: *Pme* I restriction site						
italic: overlapping sequence used in fusion PCR						
pJET1-2for	CGACTCACTATAGGGAGAGCGGC	Sequencing PglaA-cut-HIS-TglaA				ThermoFisherScientific
pJET1-2rev	AAGAACATCGATTTTCCATGGCAG	Sequencing PglaA-cut-HIS-TglaA				
PglaAP11f	TGATTTCCGCAACGGGAC	glaA 5' flank	712 bp			
PglaAP18r	*TGCTGAGGTGTAATGATGCTGG*	glaA 5' flank				
Fus_62_cut1_P1f	*CCAGCATCATTACACCTCAGCA*<br>ATGAAGTTCACCGCCGTCCTC	62-cut1-HIS ORF, overlap with PglaAP18r	847 bp		MA1084.2	This study
Fus_62_cut1_P2r	*GCGAAATGGATTGATTGTCTAGTGAT GGTGATGGTGATG*<br>GATAGAGGTAGCAGCAACGCG	62-cut1-HIS ORF, overlap with TglaAP21f				
Fus_62_cut1_P3f	TCTGGATTCA GGGCGTGAGT	Sequencing PglaA-62-cut1-HIS-TglaA				
Fus_62_cut5_P1f	*CCAGCATCATTACACCTCAGCA*<br>ATGAAGTTCTCCATCATCTCTAC	62-cut5-HIS ORF, overlap with PglaAP18r	808 bp		MA1085.2	This study
Fus_62_cut5_P2r	*GCGAAATGGATTGATTGTCTAGTGAT GGTGATGGTGATG*<br>GGCGGCTCCAGCAGCATCAG	62-cut5-HIS ORF, overlap with TglaAP21f				

Fus_62_cut5_P3f	ACAGCTCGAG GCCAAGTACG	Sequencing PglaA-62-cut5-HIS-TglaA				
Fus_38_cut1_P1f	*CCAGCATCATTACACCTCAGCA* ATGAAGTTCACCGCCGTCCTC	38-cut1-HIS ORF, overlap with PglaAP18r	884 bp		MA1086.6	This study
Fus_38_cut1_P2r	*GCGAAATGGATTGATTGTCTAGTGAT GGTGATGGTGATG* GATAGAGGTAGCAGCAACGCGCTG	38-cut1-HIS ORF, overlap with TglaAP21f				
Fus_38_cut1_P3f	GGCAGCAATG GAGTCTGGAT	Sequencing PglaA-38-cut1-HIS-TglaA				
Fus_62_cut5_P1f	*CCAGCATCATTACACCTCAGCA* ATGAAGTTCTCCATCATCTCTAC	38-cut5-HIS ORF, overlap with PglaAP18r	816 bp		MA1089.3	This study
Fus_62_cut5_P2r	*GCGAAATGGATTGATTGTCTAGTGAT GGTGATGGTGATG* GGCGGCTCCAGCAGCATCAG	38-cut5-HIS ORF, overlap with TglaAP21f				
Fus_62_cut5_P3f	ACAGCTCGAG GCCAAGTACG	Sequencing PglaA-38-cut5-HIS-TglaA				
TglaAP21f	*CATCACCATCACCATCACTAGACAATCAAT CCATTTCGC*	glaA 3' flank	746 bp			
TglaAP9r	TGGAGCCGATCAGACCAGTAG	glaA 3' flank				
PglaAP19f_PmeI	ATAAGAAT<u>GTTTAAAC</u>TCCGGACGGTCAG GAACTT	Fusion PCR PglaA-62-cut1-HIS-TglaA		2020 bp		
TglaAP23r_PmeI	ATAAGAAT<u>GTTTAAAC</u>CCGACATTCCAGCA ATACTGC	Fusion PCR PglaA-62-cut5-HIS-TglaA		1981 bp		
		Fusion PCR PglaA-38-cut1-HIS-TglaA		2057 bp		
		Fusion PCR PglaA-38-cut5-HIS-TglaA		1989 bp		

**Construction of KORE gRNA in pFC332**						Arentshorst et al., 2023
underlined: *Pac* I restriction site						
**bold: gene specific sgRNA target, overlapping sequence used in fusion PCR**						
pTE1for	CCTTAATTAAACTCCGCCGAACGTACTG	5' gRNA				
pTE1rev	CCTTAATTAAAAAAGCAAAAAAGGAAGGTACAAAAAAAGC	3' gRNA				
KORE2_sgRNA_rv	**TCATAGGTATGTAATTCTTG**GACGAGCTTACTCGTTTCGT	KORE2 5' gRNA	264 bp	377 bp		
KORE2_sgRNA_fw	**CAAGAATTACATACCTATGA**GTTTTAGAGCTAGAAATAGC	KORE2 3' gRNA	133 bp			
KORE3_sgRNA_rv	**TAATGAGGACTGTGAACAAA**GACGAGCTTACTCGTTTCGT	KORE3 5' gRNA	264 bp	377 bp		
KORE3_sgRNA_fw	**TTTGTTCACAGTCCTCATTA**GTTTTAGAGCTAGAAATAGC	KORE3 3' gRNA	133 bp			

**Diagnostic PCR to confirm integration into *A. niger* genome**						Arentshorst et al., 2023
**dPCR on NRRL3_10267 locus**						
10267_P9f	CATCGTTTCTGCACCGACC	Diagnostic PCR to confirm integration	2208 bp	62-cut1-His on 10267 locus	MA1084.2	
10267_P10r	TGAACCTGAAGCGATGGGATA	Diagnostic PCR to confirm integration	2169 bp	62-cut5-His on 10267 locus	MA1085.2	
			2245 bp	38-cut1-His on 10267 locus	MA1086.6	
			2177 bp	38-cut5-His on 10267 locus	MA1089.3	
**dPCR on *goxC* (NRRL3_02841) locus**						
goxC_P25f	GCAACCAGCCTTTCCTCTCTC	Diagnostic PCR to confirm integration	2048 bp	62-cut1-His on *goxC* locus	MA1084.2	
goxC_P26r	AGAGGATGCGCAGAGTTGCT	Diagnostic PCR to confirm integration	2009 bp	62-cut5-His on goxC locus	MA1085.2	
			2085 bp	38-cut1-His on *goxC* locus	MA1086.6	
			2017 bp	38-cut5-His on goxC locus (NO integration)	MA1089.3	
**dPCR on *oahA* (NRRL3_06354) locus**						

oahA_P17f	TGGTCACCTTCTGGCCCTT	Diagnostic PCR to confirm integration	2176 bp	62-cut1-His on *oahA* locus	MA1084.2	
oahA_P18r	TTCAATCCCATCCAATGCAGT	Diagnostic PCR to confirm integration	2137 bp	62-cut5-His on oahA locus	MA1085.2	
			2213 bp	38-cut1-His on *oahA* locus	MA1086.6	
			2145 bp	38-cut5-His on oahA locus	MA1089.3	
**dPCR on NRRL3_06629 locus**						
6629_P9f	TTCTCGAGTGGCTTGTGGTG	Diagnostic PCR to confirm integration	2368 bp	62-cut1-His on 6629 locus **(NO integration)**	MA1084.2	
6629_P10r	ACGACATCCTGCTGAGGCA	Diagnostic PCR to confirm integration	2329 bp	62-cut5-His on 6629 locus	MA1085.2	
			2405 bp	38-cut1-His on 6629 locus	MA1086.6	
			2337 bp	38-cut5-His on 6629 locus	MA1089.3	

Supplementary Table 5: **Para-nitrophenyl substrates with different tail lengths used in this study**

Catalogue name	Tail length	Cas-number	Product number
4-Nitrophenyl butyrate	C4	2635-84-9	N9876
4-Nitrophenyl octanoate	C8	1956-10-1	21742
4-Nitrophenyl decanoate	C10	1956-09-8	N0252
4-Nitrophenyl dodecanoate	C12	1956-11-2	61716
4-Nitrophenyl Myristate	C14	14617-85-7	70124
4-Nitrophenyl Palmitate	C16	1492-30-4	N2752

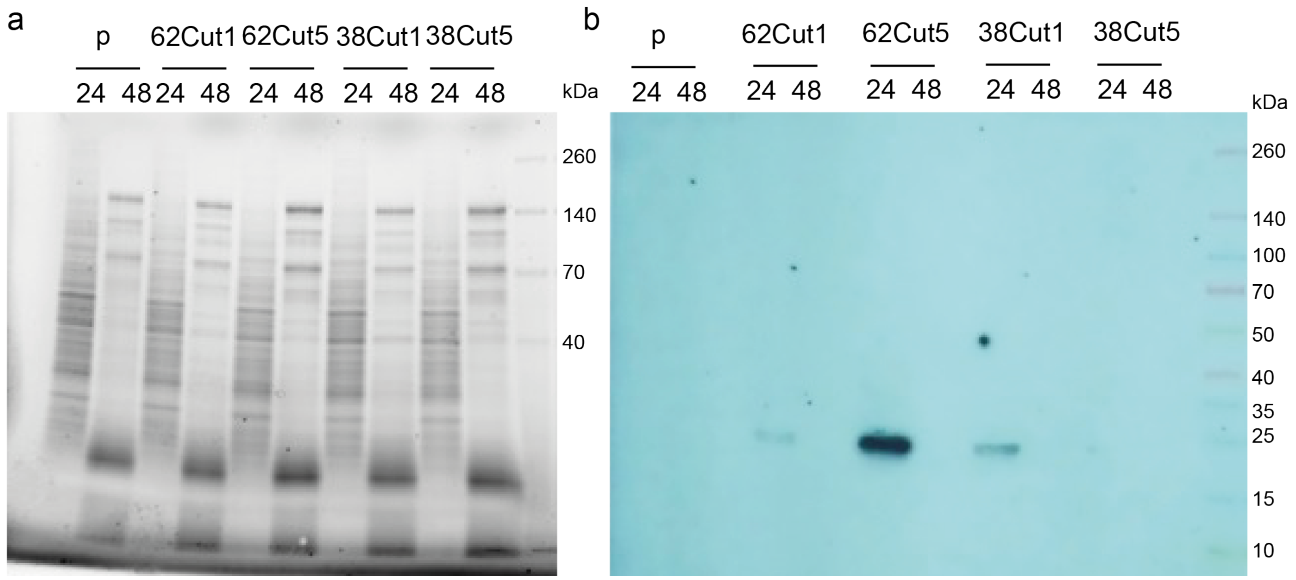

Supplementary Figure 1: ***Mycelial samples expression Cut1 and Cut 5***

*a) SDS-PAGE of 24 h and 48 h cultivation samples of parental strain (p), Fr*62Cut1 (62Cut1), *Fo*38Cut1 (38Cut1), *Fr*62Cut5 (62Cut5) and *Fo*38Cut5 (38Cut5). b) Western blot *of 24 h and 48 h cultivation samples of parental strain (p), Fr*62Cut1 (62Cut1), *Fo*38Cut1 (38Cut1), *Fr*62Cut5 (62Cut5) and *Fo*38Cut5 (38Cut5).
